# Not All Heart Attacks are Created Equal: Thinking Differently About Acute Myocardial Infarction in the Young

**DOI:** 10.14797/mdcvj.345

**Published:** 2021-09-24

**Authors:** Theresa Rizk, Ron Blankstein

**Affiliations:** 1 Duke University School of Medicine, Durham, North Carolina, US; 2 Brigham and Women’s Hospital, Brookline, Massachusetts, US; 3 Harvard Medical School, Cambridge, Massachusetts, US

**Keywords:** acute myocardial infarction, adults, coronary atherosclerosis, risk factors, sex differences, youth

## Abstract

Cardiovascular disease, particularly myocardial infarction, remains a major cause of morbidity and mortality among young individuals. Although myocardial infarctions have declined significantly in the general population, this decline has not been uniformly observed in younger cohorts. Young adults often have different risk factors, including a higher burden of tobacco use and substance abuse, and they are less likely to be treated with preventive therapies since they are often categorized as having low risk. This review examines the existing literature on myocardial infarction in young patients, with a focus on risk factors, therapeutic challenges, and opportunities for prevention.

## Introduction

Mortality rates from cardiovascular disease (CVD) have declined in the last few decades due to continued advancements in the field of cardiovascular medicine. In addition, the proportion of deaths attributed to ischemic heart disease decreased from 73% in 1999 to 56% in 2018. However, a recent update from the American Heart Association (AHA) indicates that CVD remains prevalent in nearly half of the US population over 20 years of age and in 25% of young adults aged 20 to 39 years.^1^ While the incidence of acute coronary syndrome (ACS) in older adults is declining, hospitalization rates for young people with acute myocardial infarction (MI) have not demonstrated a similar trend.^2^ Possible factors that may be contributing to these differences include under-recognition of the risks in younger populations and failure to identify and treat various risk factors. Another factor may be age; although studies vary widely in their definition of “young,” many articles define it as under age 55 and some use even younger cutoffs.^3^ In this review, we summarize the existing literature on myocardial infarction in young patients with a focus on risk factors, therapeutic challenges, and opportunities for prevention.

## Methods

We conducted a literature search via PubMed for the years 1980 to 2020 using the keywords “myocardial infarction,” “acute myocardial infarction,” “young,” “young adults,” and the MeSH terms “MI/etiology,” “MI/mortality,” and “age factors.” The search was limited to papers published in English and human studies, excluding case reports. ***Table 1*** summarizes the included studies.

**Table 1:** A summary of the studies discussed in this review. M: males; F: females; y: years; MI: myocardial infarction; AMI: acute myocardial infarction; Lp(a): lipoprotein a; ACS: acute coronary syndrome; CAD: coronary artery disease; CVD: cardiovascular disease; Hx: history; N/A: not applicable.


AUTHOR	YEAR	JOURNAL	TYPE	POPULATION

Akosah KO	2003	*Journal of the American College of Cardiology*	Retrospective cohort	M < 55 y, F < 65 y hospitalized for acute MI (mean 50)

Anderson RE	2008	*American Heart Journal*	Retrospective cohort	Patients aged 18–45 (803), 45–65 (6185), > 65 y (7715) with MI

Arora S	2019	*Circulation*	Retrospective cohort	8,737 patients aged 35–54 y with AMI

Berman AN	2020	*European Journal of Preventive Cardiology*	Retrospective cohort	Patients aged < 50 y with MI, 441 with Lp(a) measured

Biery DW	2020	*JAMA Network Open*	Retrospective cohort	2,072 YOUNG-MI patients aged < 50 y with MI

Bucholz EM	2017	*European Heart Journal – Acute CV Care*	Retrospective cohort	3,501 patients aged < 55 y with AMI

Canto JG	2000	*JAMA*	Prospective cohort	434,877 patients with MI

Choudhury L	1999	*American Journal of Medicine*	Review	N/A (literature review – see reference 13)

Davidson L	2014	*American Journal of Medicine*	Retrospective cohort	124 patients aged ≤ 35 y with ACS

DeFilippis EM	2018	*Journal of the American College of Cardiology*	Retrospective cohort	2,097 patients aged < 50 y with MI

DeFilippis EM	2020	*European Heart Journal*	Retrospective cohort	2,097 patients aged < 50 y with MI

Divakaran S	2020	*Diabetes Care*	Retrospective cohort	2,097 YOUNG-MI patients aged < 50 y with MI

Doughty M	2002	*American Heart Journal*	Retrospective cohort	976 patients aged < 46, 46–54, and > 54 y with MI

Egiziano G	2013	*Diabetic Medicine*	Retrospective cohort	10,619 MI survivors aged < 50 y

Fournier JA	2004	*American Journal of Cardiology*	Prospective cohort	104 patients aged ≤ 40 y with MI followed for 15 y

Garshick	2019	*Cardiology*	Retrospective cohort	281 (aged 50 +/– 6 y) vs 799 (aged 69 +/– 7.5 y) patients with CAD

Gulati R	2020	*Mayo Clinic Proceedings*	Review	N/A (see reference 3)

Gupta A	2014	*Journal of the American College of Cardiology*	Retrospective cohort	230,684 patients aged 30–54 y with AMI

Kannel WB	1990	*Advanced Cardiology*	Prospective cohort	N/A (see reference 10)

McManus DD	2011	*American Journal of Cardiology*	Retrospective cohort	1,703 patients aged 25–54 y with MI

Miedema MD	2019	*JAMA Network Open*	Retrospective cohort	22,346 adults aged 30–49 y without CVD

Moccetti T	2007	*Archives of Internal Medicine*	Retrospective cohort	11,483 patients aged < 50, 50–70, > 70 y with MI

Pineda J	2008	*International Journal of Cardiology*	Retrospective cohort	200 patients aged < 45 y and 200 > 45 y with coronary disease

Schoenenberger AW	2011	*International Journal of Cardiology*	Prospective cohort	195 patients aged < 35 y with ACS

Singh A	2017	*Clinical Cardiology*	Study description	N/A (see reference 20)

Singh A	2018	*Journal of the American College of Cardiology*	Retrospective cohort	1,685 patients with MI

Singh A	2019	*Journal of the American College of Cardiology*	Retrospective cohort	1,996 patients aged < 50 y with MI

Singh A	2020	*Journal of the American College of Cardiology*	Retrospective cohort	3,829 patients aged < 50 y with MI

Vikulova DN	2019	*Journal of the American Heart Association*	Retrospective cohort	12,519 patients with CAD (70% M < 50 y, 30% F < 55 y)

Virani SS	2020	*Circulation*	Statistical report	N/A (see reference 1)

Wiesbauer F	2009	*European Heart Journal*	Prospective cohort	102 MI survivors aged ≤ 40 y

Wu WY	2020	*Journal of the American College of Cardiology*	Retrospective cohort	1,724 patients aged < 50 y with MI

Zeitouni M	2020	*Journal of the American College of Cardiology*	Retrospective cohort	Patients aged < 55 y with MI

Zimmerman FH	1995	*Journal of the American College of Cardiology*	Retrospective cohort	294 M, 210 F with Hx MI


## Definitions and Prevalence

With respect to premature MI and coronary heart disease (CHD), there is a wide discrepancy in the literature regarding the definition of “young,” with studies varying between < 35 years^4,5^ to < 55 years^6,7^ and some setting different cutoffs for men versus women.^8,9^ Given these discrepancies, we discuss the relevant age cutoffs used in individual studies rather than using a single definition.

Recent statistics show that CVD remains the country’s leading cause of death, with an age-adjusted death rate of 200.8 per 100,000 population.^1^ And while the burden of US deaths attributable to cardiovascular diseases has declined over the past two decades, there is no corresponding decrease in acute MI hospitalizations in those aged 55 years and younger.^2^ Overall, the prevalence of acute MI among young patient populations varies according to age cutoff and the definition of MI, and it is difficult to determine accurately given the limited data. However, the Framingham Heart Study published a 10-year follow-up survey of individuals in three age categories (30 to 34, 35 to 44, and 45 to 54 years), indicating the incidence of MI in each age group to be 12.9, 38.2, and 71.2 per 1,000 men and 2.2, 5.2, and 13.0 per 1,000 women, respectively.^10^ A study by McManus et al. reports an MI incidence of only 66 per 100,000 patients ages 25 to 54 years, with incidence rates decreasing inconsistently over time,^11^ whereas Doughty et al. demonstrated that > 10% of all patients presenting with MI at the University of Michigan Medical Center (UMMC) were < 46 years of age.^12^

## Risk Factors and Presentation

Many studies have sought to examine the risk factors that appear to contribute uniquely to the risk of MI in younger populations. Tobacco use, family history of premature CVD, and hyperlipidemia are often the leading risk factors, followed by contributions from substance abuse, diabetes, psychological factors, and socioeconomic status (SES). Interestingly, while many of these risk factors were first described decades ago as highly prevalent in the young, they remain equally prevalent in more contemporary studies. In 1999, for example, Choudhury et al. observed that among individuals < 45 years of age, acute MI is predominantly present in males and most strongly associated with a family history of MI before the age of 55, hyperlipidemia, smoking, or obesity.^13^ In the VALIANT (Valsartan in acute myocardial infarction) trial, which studied 14,703 individuals with acute MI complicated by heart failure or systolic dysfunction, Anderson et al. found that those aged 18 to 45 were less likely than their older counterparts to present with a history of diabetes mellitus (DM), hypertension, or prior MI and more likely to be non-White males and current smokers and to have a history of obesity and dyslipidemia.^14^

Similarly, Pineda et al. in 2008 showed that individuals < 45 years old with coronary disease (defined as one or more coronary lesions of ≥ 70% stenosis) were more likely than older patients to be male, smokers, and suffer from hyperlipidemia, often presenting with less extensive coronary atherosclerosis and a higher incidence of single-vessel disease.^15^ In addition, Garshick et al. observed that younger patients (mean age 50 ± 6 years) undergoing coronary intervention for obstructive coronary artery disease (CAD) experienced higher rates of psychological and financial stressors and lower overall functional capacity than older patients (mean age 69 ± 7.5 years).^16^ A computer simulation study of 1.3 million 35-year-olds with low SES projected that their risk of developing coronary heart disease by age 65 was nearly double the rate projected for individuals of higher SES.^17^ The authors therefore stressed the importance of addressing low SES (low income and/or low education level) as a risk factor for premature heart disease.

Several studies have emphasized that tobacco and drug use are some of the most important modifiable risk factors for MI among younger individuals. For instance, Zimmerman et al. found that current smoking was more frequent in young patients (men ≤ 35 years and women ≤ 45 years) with a history of MI compared to their older counterparts (*P* < .0001).^9^ Similarly, Doughty et al. found that individuals who experienced an MI before the age of 46 were more likely to have a family history of premature heart disease and tobacco use, emphasizing the benefit of addressing smoking cessation and cardiac rehabilitation among younger individuals.^12^ A study of substance abuse in the YOUNG-MI registry cohort found that use of cocaine or marijuana was present in 10.7% of patients and associated with significantly higher long-term CV and all-cause mortality; it also found that users had lower rates of diabetes and hyperlipidemia but were significantly more likely to use tobacco.^18^

Inherited dyslipidemias are also prevalent risk factors among young adults who experience an MI. For instance, familial-combined hyperlipidemia (FCHL) has been associated with a 24-fold increased adjusted risk for MI (95% CI, 7.5–81; *P* < .001) in individuals ≤ 40 years of age.^19^ Similarly, Singh et al. examined the Partners YOUNG-MI registry for the prevalence of familial hypercholesterolemia (FH) and found that clinically defined FH was identified in nearly 10% of patients experiencing an MI before the age of 50.^20^ Another lipid emerging as an important risk factor in the young population is lipoprotein(a), or Lp(a).^21^ In a separate study of the YOUNG-MI cohort, Singh et al. observed that one in three patients < 50 years of age who presented with MI had an Lp(a) level above the 80th percentile.^22^

**Figure 1:**
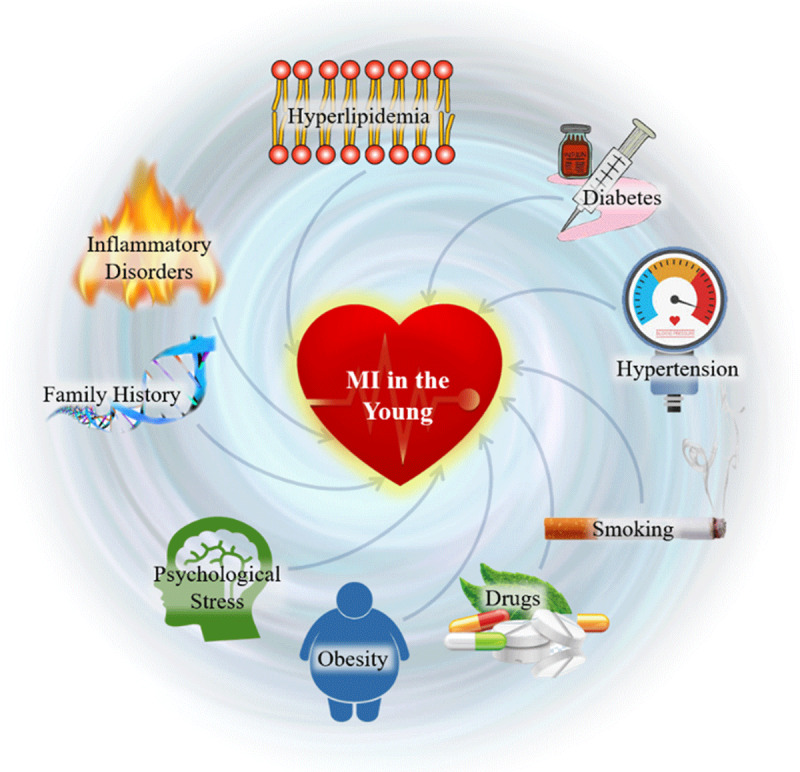
Both traditional and novel risk factors contribute to the development of premature MI in younger populations. MI: myocardial infarction.

In summary, traditional risk factors such as hypertension, male sex, obesity, and hyperlipidemia all contribute to the risk of MI at a young age, but additional risk factors such as a family history of premature CHD, smoking, substance abuse, diabetes, and psychological stressors have also been uniquely shown to contribute to the risk profile of younger individuals (Figure 1).

## Clinical Presentation

Individuals who experience an MI at a young age can also present with atypical clinical and angiographic findings. Egiziano et al. observed that only 25% of patients < 50 years who experienced an MI reported chest pain in the month prior to their acute event (a proportion that was even lower in women^23^) compared with 67% who reported chest pain in the general MI population.^24^ However, in the YOUNG-MI cohort, DeFillipis et al. found that chest pain was the most common presenting symptom in both men (90%) and women (88%) at the time of their event and presentation to the hospital.^25^ The study by Zimmerman et al., which evaluated angiographic characteristics in men ≤ 35 years and women ≤ 45 years who experienced an MI, found that younger patients more often had angiographically normal coronary arteries, nonobstructive disease (< 70% stenosis), and single-vessel disease than the older population (*P* < .0001).^9^ In evaluating 108 patients ≤ 40 years who experienced an acute MI, Fournier et al. similarly found that coronary arteries were angiographically normal in 20% of them, while three-vessel disease was reported in only 10%.^26^

## Prevention and Management

A review of the literature shows that current CVD prevention guidelines underestimate risk in younger individuals and that current algorithms fail to identify many at-risk young individuals. Akosah et al. investigated the effectiveness of the National Cholesterol Education Program (NCEP) III guidelines in identifying young adults at risk for a cardiac event. In a cohort of men ≤ 55 years and women ≤ 65 years who were hospitalized for acute MI, they found that only 25% qualified for pharmacotherapy based their 10-year risk, with an even smaller proportion of women qualifying.^6^ They also proposed that the guidelines may underappreciate the risk for disease in a younger population. Similarly, Singh et al. examined the YOUNG-MI registry to evaluate statin eligibility in adults who experienced a first-time MI at a young age.^27^ Out of 1,475 patients who experienced a type 1 MI under age 50, 83% reported one or more cardiovascular risk factors, yet their median 10-year ASCVD risk was only 4.8%, which meant that only 49% of patients would have been eligible for statin treatment based on the 2013 American College of Cardiology/American Heart Association guidelines and 2016 US Preventive Services Task Force recommendations. The guidelines had a greater impact on women, in that 63% would have been ineligible for statin therapy prior to their MI. The following year, Singh et al. examined the same cohort for the management of FH and found that 42.8% of patients with clinically defined FH were not on statin therapy prior to their MI. When evaluating post-MI care, only 63.3% were discharged on high-intensity statin therapy, and 82.2% had elevated LDL-C at 1-year follow-up.^22^ Zeitouni et al. compared the 2013 and 2018 ACC/AHA cholesterol guidelines for their applicability to young adults aged < 55 years with premature MI and found that intensive post-MI lipid management was not recommended for 71.7% of younger individuals.^28^

## Prognosis/Outcomes

Outcomes are generally more favorable in patients experiencing an acute MI at a young age but often depend on smoking status, lifestyle factors, and comorbidities. In a study by Moccetti et al. that evaluated 11,483 individuals with MI according to their age, age < 50 years was a significant independent predictor of lower in-hospital and 6-month mortality compared with older age groups.^29^ A prospective cohort study by Fournier et al. of individuals ≤ 40 years old who experienced an MI found that type I DM, excessive alcohol intake, peripheral arterial disease (PAD), previous MI, and decreased left ventricular ejection fraction (LVEF) were predictors of increased mortality following premature MI, with the strongest predictors being LVEF ≤ 45% and PAD.^26^

Several recent studies from the YOUNG-MI registry have also revealed patterns in the prognosis of young adults (< 50 years) after MI. Biery et al. found that smoking cessation within 1 year after MI was associated with significantly lower long-term all-cause mortality and cardiovascular mortality.^30^ In a study by Wu et al., 42% of patients with an abnormal LVEF after MI recovered to an EF ≥ 50%, which was associated with an 8-fold reduction in all-cause mortality and 10-fold reduction in cardiovascular mortality.^31^ Divakaran et al. showed that among individuals who had an MI at a young age, the presence of diabetes was associated with higher all-cause mortality (HR 2.30; *P* < .001) and cardiovascular mortality (HR 2.68; *P* < .001) over a mean follow-up of 11.2 years.^32^ Yang et al. looked more closely at individuals from this cohort aged ≤ 40 years and found that they experienced similar rates of all-cause and CV mortality compared to those aged 41 to 50 years and thus did not appear to be protected by an average age difference of 10 years.^33^

## Sex Differences

The differences between men and women regarding management and outcome following MI has garnered more attention in recent years, particularly in the younger population. Several studies have illustrated a trend in which the prevalence of comorbidities is higher in women who experience an MI under age 55. Compared with men, women also had longer in-hospital length of stay, were less likely to undergo coronary revascularization, and had a higher long-term all-cause mortality following MI.^2,7,8^ An important study by Arora et al. noted that the overall incidence of hospitalizations for acute MI increased for young women (35–54 years old) from 1995 to 2014 but decreased in young men; furthermore, young women had a greater comorbidity burden and were less likely to receive lipid-lowering therapy, antiplatelets, beta blockers, coronary angiography, and revascularization procedures.^34^ However, all-cause mortality after 1 year was comparable in women versus men. Similarly, DeFillipis et al. found that women in the YOUNG-MI registry were more likely to have diabetes and less likely to undergo angiography or be discharged on aspirin, beta blockers, angiotensin-converting enzyme inhibitors/angiotensin receptor blockers, and statins.^25^

## Coronary Artery Calcium Testing

Coronary artery calcium (CAC) testing can detect the presence and severity of calcified coronary plaque and is useful when there is uncertainty regarding patient risk. While current guidelines provide a role for CAC testing over age 40, emerging data suggests that selective use of CAC may also be useful among younger individuals. Miedema et al. examined the association between elevated CAD and premature CHD among adults ages 30 to 49 years and found that 34% of those referred for CAC testing had atherosclerosis, although only 7% had a CAC score > 100.^35^ Notably, a CAC score > 100 was associated with a marked increased risk of CHD (HR 5.6; 95% CI, 2.5–12.7), CVD (HR 3.3; 95% CI, 1.8–6.2), and all-cause mortality (HR 2.6; 95% CI, 1.9–3.6) compared to those with a CAC score of 0. This study supports the fact that the presence of any plaque at a young age is indicative of higher risk and should prompt more aggressive pharmacotherapy and lifestyle changes. It is also important to note that the absence of CAC at a young age should not always be viewed as reassuring since coronary plaque may have not calcified. Thus, treatment of underlying risk factors remains important in all at-risk young adults.

## Recommendations

There is an unmet need for improved risk assessment among young individuals. Future studies should evaluate whether this can be achieved by recalibrating existing risk calculators to account for factors that may be more prevalent and impactful in this age group or if new risk scores—possibly integrating data on polygenic risk or CAC—are needed. Greater emphasis should be placed on identifying existing cardiovascular risk factors rather than relying on risk scores based on age. Lifestyle therapies should be addressed sooner and more proactively in the young, including tobacco cessation, weight loss, adopting a healthy diet, and regular exercise. Selective use of CAC scores can identify individuals with premature atherosclerosis and prompt earlier initiation of lipid-lowering therapy and other interventions. Finally, physicians should routinely ask patients if they have a family history of premature heart disease. Although they require further validation, polygenic risk scores also represent a promising approach for identifying at-risk individuals and informing clinical management.

## Conclusion

Prevention of MI in young individuals is an important public health problem. Despite being categorized as “low risk” prior to their events, most young individuals who experience an MI have pre-existing risk factors, such as obesity, diabetes, hypertension, and hyperlipidemia. Tobacco use, which occurs in approximately 50% of young adults who experience an MI, remains one of the most important modifiable risk factors. Additionally, substance abuse, tobacco use, diabetes, left ventricular systolic dysfunction, and systemic inflammatory disease are all associated with a worse long-term prognosis in those who experience an MI at a young age. These findings have important implications for both primary and secondary prevention.

## Key Points

- Cardiovascular (CV) disease remains prevalent in nearly half of the US population over age 20 and in 25% of young adults aged 20 to 39 years.
- Young individuals have not experienced the same decline in CV mortality as their older counterparts.
- Young adults who experience a myocardial infarction (MI) have risk factors unique to their age group.
- Risk calculators used by current guidelines may underestimate cardiovascular risk in young adults. As a result, young adults often do not meet guideline indications for lipid-lowering therapies.
- Risk factors and outcomes differ between young men and women who experience an MI.
- Short-term outcomes are relatively favorable among young patients post MI, but long-term prognosis is significantly impacted.

## CME Credit Opportunity

Houston Methodist is accredited by the Accreditation Council for Continuing Medical Education (ACCME) to provide continuing medical education for physicians.

Houston Methodist designates this enduring material for a maximum of .25 *AMA PRA Category 1 Credit*™. Physicians should claim only the credit commensurate with the extent of their participation in the activity.

Click to earn CME credit: https://cvent.me/QrGqMZ.
